# The impact of how physicians self‐present: A study of gender and attire effects on perceived warmth and competence

**DOI:** 10.1111/bjhp.70054

**Published:** 2026-01-26

**Authors:** Hannah Meltser, David M. Markowitz

**Affiliations:** ^1^ Department of Communication Michigan State University East Lansing Michigan USA

**Keywords:** competence, medicine, self‐presentation, trust, warmth

## Abstract

**Objectives:**

We aimed to replicate the idea that physicians' self‐presentation characteristics (i.e. formal vs. informal attire) and gender (i.e. men vs. women) influence perceptions of their warmth and competence. Further, we aimed to extend this line of work by examining how these relationships are moderated by trust in physicians.

**Design:**

We conducted a 2 (physician gender: men vs. women) × 2 (self‐presentation: formal vs. informal attire) experiment using publicly available physician images.

**Methods:**

Students (*N* = 734) were randomly assigned to rate five physician images from a pool of 20 stimuli across physician gender and attire conditions. Participants rated physicians on warmth and competence, and then completed a trust in physicians scale. Linear mixed models evaluated main effects and interaction effects for self‐presentation, gender and physician trust.

**Results:**

Physicians in formal attire (white lab coats) were perceived as warmer and more competent than those in informal attire (casual or informal wear). Women physicians were rated as warmer, but not more competent than men physicians. Two‐way interaction effects revealed formal attire enhanced perceptions of men physicians more than women on average. Three‐way interaction effects indicated trust in physicians moderated these results, with women physicians' ratings being more dependent on participants' general trust levels, particularly for those with lower trust in the medical profession.

**Conclusions:**

We replicated and extended this literature by demonstrating how physician gender and patient trust levels moderate self‐presentation effects. For physicians, understanding a patient's relationship with the medical establishment may help to inform their self‐presentation choices.

**Statement of Contribution**

**What is already known on this subject?**

- Perceptions of physicians are often impacted by self‐presentation aspects of the caregiver.
- Patients often prefer physicians in formal and professional attire over informal and casual attire.

**What does this study add?**

- This work provides a necessary replication attempt of prior studies to evaluate how physicians in formal versus informal attire are perceived.
- This work extends prior scholarship by examining how self‐presentation (i.e. formal vs. informal attire), gender (i.e. men vs. women) and physician trust jointly link to physician perceptions such as warmth and competence.

## INTRODUCTION

People form impressions about physicians and their health care providers using a range of communication data. For example, people routinely make judgements about physicians based on facial features of the caregiver (Mattarozzi et al., 2017), their physical characteristics (Hall & Ruben, 2020) and emotional displays (Senft et al., 2018), which can impact how much trust and confidence patients have in such caregivers (Hillen et al., 2011; Jongerius et al., 2022; Yang et al., 2024). Impressions of physicians are critical because people can use this interpersonal information to inform how they think and feel about the medical system, in general (Pearson & Raeke, 2000). Recent evidence suggests impressions of physicians and medical scientists are deteriorating over time with little indication of deceleration (Kennedy & Tyson, 2023). Against this disheartening backdrop, it is therefore critical to evaluate how physicians might be perceived by patients as a function of caregivers' social and psychological characteristics, which can help to inform how impression formation occurs in this consequential setting.

Specifically, this paper draws on self‐presentation theory (Goffman, 1959, 1967) and person‐perception research (Fiske et al., 2002) to evaluate how physician characteristics impact how people form impressions about such caregivers. We evaluated how nonverbal aspects of physicians' self‐presentation (i.e. their formal or informal attire) and physician gender (i.e. men versus women) impact how people perceive their warmth and competence. This work is timely and important because it provides a necessary replication and extension of prior work that has investigated how people make judgements about physicians based on nonverbal (e.g. attire‐related) characteristics. We address an opportunity to examine how first impressions of caregivers are impacted by two critical and often conflated factors in experimental research: self‐presentation and identity. Here, we provide experimental evidence that suggests people form more positive impressions of physicians based on nonverbal characteristics like how they are dressed and that physician ratings differ as a function of both self‐presentation and gender. We also explore how warmth and competence are jointly impacted by self‐presentation, gender and trust in physicians, which offers a novel contribution to the literature by examining how individual differences via trust perceptions shape physician ratings.

### An overview of self‐presentation theory

Landmark research by Goffman (1959) argued that a fundamental human need is for people to manage how they appear to others. Thus, people often regulate how they self‐present to achieve various social, psychological and communicative goals (Baumeister, 1982; Snyder, 1974). People often want to self‐present positively in order to appear trustworthy (Kim et al., 2023), warm and competent (Fiske, 2018; Rom & Conway, 2018) and modest to others (Robinson et al., 1995). This is facilitated by self‐presentation strategies such as idealization (e.g. amplifying how one appears; Sun et al., 2021) or those that downplay one's individualized characteristics in order to blend in (Rui & Stefanone, 2013). Across many ways that people might self‐present verbally or nonverbally (DePaulo, 1992; Walther, 2007), an overarching interest remains: People use self‐presentation as a means for achieving impression management ends that can help to facilitate communication goals (Hollenbaugh, 2021).

Impression management is particularly important in consequential and high‐stakes interpersonal settings such as medicine. Indeed, a range of evidence suggests physicians try to present professionally online (Maggio et al., 2024), appear like competent and effective practitioners (Cantillon et al., 2021; Huffman et al., 2021; Molloy & Bearman, 2019) and be perceived as approachable to patients (Atef et al., 2023). How can physicians achieve such positive self‐presentation goals? Recent evidence indicates physicians' self‐presentation can be amplified via verbal and nonverbal means. For example, Markowitz (2023) found that physicians who communicated more personally (e.g. with more self‐references) and confidently (e.g. using a high rate of certainty terms and low rate of tentativeness terms) in their HealthGrades profiles were more positively rated than physicians who communicated less personally and less confidently. In a follow‐up experiment, physicians who communicated with more ‘I’‐words and certainty terms were rated as warmer and more competent than physicians with fewer of these verbal characteristics in their profiles, demonstrating the potential for physician language patterns to impact how patients think and feel about them. Nonverbally, an important line of work has analysed how physician attire symbolizes cultural capital, rank, formality and professionalism (Jenkins, 2014). Evidence suggests patients often prefer formal and professional attire for physicians like a white lab coat over scrubs, business attire or casual attire (Rehman et al., 2005). That is, physicians in a white lab coat were more likely to be perceived as trustworthy than those in other attire, and patients were more likely to disclose medical problems with physicians who dressed formally. This general finding, where patients prefer professional and formal attire over casual and informal attire, has been replicated in other medical settings largely via survey methods (Ahmed et al., 2020; Au et al., 2013; Petrilli et al., 2018; Xun et al., 2021).

### The current study

Altogether, there are downstream consequences for physicians based on their verbal and nonverbal self‐presentation acts. The goal of the current research is to build on and extend existing self‐presentation and medicine research by experimentally testing how nonverbal aspects of physicians' self‐presentation—specifically, formal (operationalized as a white lab coat) versus informal attire (operationalized as business attire)—impact warmth and competence perceptions. Our interest in warmth and competence stems from prior research that suggests people are systematically adept at making such perceptions about people across a range of settings (Fiske, 2012, 2018; Fiske et al., 2002), especially in medicine (Drevs, 2013; Kraft‐Todd et al., 2017; Markowitz, 2023). Attire is an integral part of person‐perception and first impressions (Hareli et al., 2025; Kurihara et al., 2014), and patient experiences may be impacted by what they deem as most warm or competent in a physician's appearance. Therefore, in one important sense, our work provides a necessary replication attempt of prior studies to evaluate how physicians in formal versus informal attire are judged. Replications in the social and psychological sciences are rare (e.g. Keating & Totzkay, 2019; Markowitz et al., 2021), but critical to the validity of our theories and prior empirical evidence.

Crucially, we also wanted to identify how physician demographic characteristics, namely gender, might moderate prior attire effects. This is critical because physician ratings tend to be moderated by or systematically linked to gender (Madanay et al., 2025; Markowitz, 2023), and prior work has either overlooked or ignored how physician perceptions are moderated by this demographic characteristic, at least in experimental work. Together, our research evaluates and disentangles the joint impact of physician self‐presentation (i.e. attire effects) and gender to understand how caregiver perceptions are impacted by such factors, which leads to our overarching research question:

RQ: What is the relationship between self‐presentation, gender and their interaction to predict warmth and competence perceptions?

Our work also seeks to extend prior scholarship in one critical way. We specifically explored how individual differences in patient experiences with physicians might impact warmth and competence perceptions. Prior work suggests trust in physicians is essential to understand how people form impressions of their doctors, and it can ‘mediate medical outcomes’ like adherence to treatment (Hall et al., 2001, p. 614). For example, patients with lower trust in physicians are more likely to interpret ambiguous clinical behaviours as signs of reduced warmth (e.g. empathy) or competence (e.g. perceived effectiveness), reflecting a general tendency for trust to guide interpersonal perceptions in health care (Birkhäuer et al., 2017). Trust also shapes broader relational expectations, as patients who report higher levels of trust with physicians tend to perceive their doctors as more empathetic, communicative and effective compared to those with lower levels of trust with physicians (Thom et al., 2004). Trust in physicians is therefore another essential characteristic we use to assess how people might form impressions of caregivers in addition to self‐presentation data and physician gender. We extend prior scholarship by examining how self‐presentation (i.e. formal vs. informal attire), gender (i.e. men vs. women), and physician trust jointly link to perceptions such as warmth and competence.

## METHOD

### Statistical power and participants

We conducted an a priori power analysis to ensure that enough participants were collected for our repeated‐measures design. Estimating a small effect size (Cohen's *f* = .10) at 80% power (*α* = .05), with four experimental groups and five repeated measurements, a total of 660 participants were required for this study. We used the Department of Communication's research participant recruitment pool at a large Midwestern university (via SONA) to collect data from 734 students. Most participants self‐identified as women (*n* = 411; 55.99%) and were 19.67 years old (SD = 2.06 years), on average. Most participants self‐identified as White (*n* = 591, 80.5%) with participants of other ethnicities being less represented in the sample (Black *n* = 54; Asian *n* = 47; Other *n* = 28; American Indian *n* = 2; Native Hawaiian *n* = 2; others did not disclose). The study received Institutional Review Board approval from Michigan State University (STUDY00011382).

### Procedure

Upon obtaining informed consent, participants were introduced to a physician perceptions study and told that they would make judgements about physicians based on images. Actual physician images that were publicly available from HealthGrades, a site used to review physicians based on personal experience, were selected by members of the research team based on their characteristics that fit the 2 (gender: men vs. women) × 2 (self‐presentation: formal vs. informal attire) experimental design. Physician ethnicity (White) and perceived age were consistent to avoid experimental confounds. Five stimuli were created for each experimental condition, which created a total of 20 stimuli in the experiment. Participants were randomly assigned to five of the 20 stimuli across experimental conditions.

After participants saw an image of a physician, they made judgements based on the physician's perceived warmth and competence in random order (Fiske, 2018; Fiske et al., 2002). Finally, participants responded to a trust in physicians scale (Hall et al., 2002), followed by basic demographic information, and then they exited the Qualtrics survey interface. Images used in this study are available from the authors upon request.

### Measures

Consistent with prior work (Fiske et al., 2002; Markowitz, 2023), participants made warmth (i.e. *friendly*, *well‐intentioned*, *trustworthy*, *warm*, *good‐natured*, *sincere*; Cronbach's *α* = .94) and competence (i.e. *competent*, *confident*, *capable*, *efficient*, *intelligent*, *skilful*; Cronbach's *α* = .94) perceptions by rating physicians on such attributes. All attributes were made on 7‐point Likert‐type scales by soliciting agreement ratings (1 = extremely disagree, 7 = extremely agree). The trust in physicians scale was an 11‐item composite (Cronbach's *α* = .86) containing agreement measures soliciting responses to items like ‘Doctors are extremely thorough and careful’ and ‘A doctor would never mislead you about anything’. Items were measured on 5‐point Likert‐type agreement statements (1 = Strongly disagree, 5 = Strongly agree), and scores were averaged to create a single measure of trust perceptions.

### Analytic plan

Due to the repeated nature of the measures collected from participants, the data were not independent and linear mixed models—with a random intercept for participant—were used to evaluate how gender, self‐presentation and their interaction impact warmth and competence (Bates et al., 2015; Kuznetsova et al., 2020). Effect sizes for fixed effects (*R* ^2^m, or marginal *R* ^2^) and fixed plus random effects (*R* ^2^c, or conditional *R* ^2^) were computed using the MuMIn package (Bartoń, 2020). In main effect analyses, both gender (men vs. women) and self‐presentation (formal vs. informal attire) were entered as simultaneous predictors. Formulae for our linear mixed models are indicated in the online supplement for transparency.

## RESULTS

Descriptive statistics and intercorrelations between key variables are in Table 1.

**TABLE 1:** Descriptive statistics and intercorrelations across key variables.

Variable	M	SD	1	2
1. Warmth	5.27	1.09	–	
2. Competence	5.46	1.00	.75** [.74, .76]	
3. Trust perceptions	3.23	.68	.22** [.18, .25]	.19** [.16, .23]

*Note*: Numbers in brackets at 95% Confidence Intervals. Correlations between variables are represented in columns after means and standard deviations. ** *p* < .01.

### Main effects

After accounting for physician gender, those presented in formal attire were perceived as warmer than those presented in informal attire (*B* = .21, SE = .029, *t* = 6.96, *p* < .001, *R* ^2^m = .024, *R* ^2^c = .436). In the same model, after controlling for attire, women physicians were perceived as warmer than men physicians (*B* = .27, SE = .030, *t* = 9.06, *p* < .001). These effects were maintained after accounting for trust perceptions.

After accounting for physician gender, those presented in formal attire were perceived as more competent than those presented in informal attire (*B* = .10, SE = .025, *t* = 4.00, *p* < .001, *R* ^2^m = .003, *R* ^2^c = .536). In the same model, after controlling for attire, there was no statistically significant relationship between physician gender and competence (*p* = .272). These effects were also maintained after accounting for trust perceptions as well.

### Interaction effects

**FIGURE 1:**
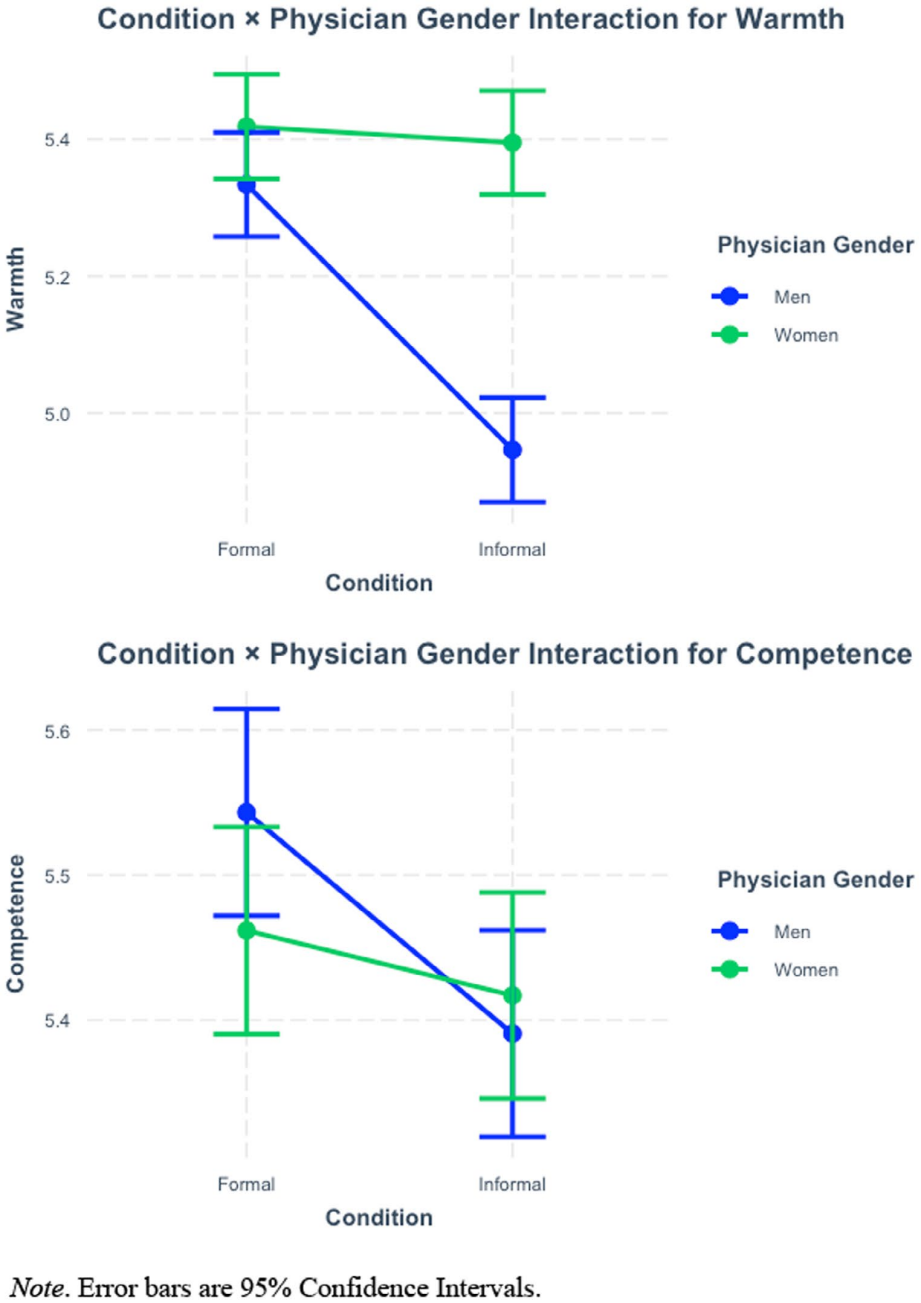
Two‐way interaction effect results. Error bars are 95% confidence intervals.

Gender (men vs. women) × Self‐Presentation (formal vs. informal attire) interaction effects were statistically significant for both warmth [*F*(1, 3052.1) = 38.69, *p* < .001] and competence [*F*(1, 2971.4) = 4.79, *p* = .029]. The images in Figure 1 display these patterns directly. For warmth, Tukey's HSD revealed men in informal attire were perceived as less warm than women in formal (*p* < .001) and informal attire (*p* < .001). Men in formal attire were also perceived as warmer than men in informal attire (*p* < .001). Finally, men in formal attire were perceived as more competent than men in informal attire (*p* < .001), and men in formal attire were perceived as more competent than women in informal attire (*p* = .002).

**FIGURE 2:**
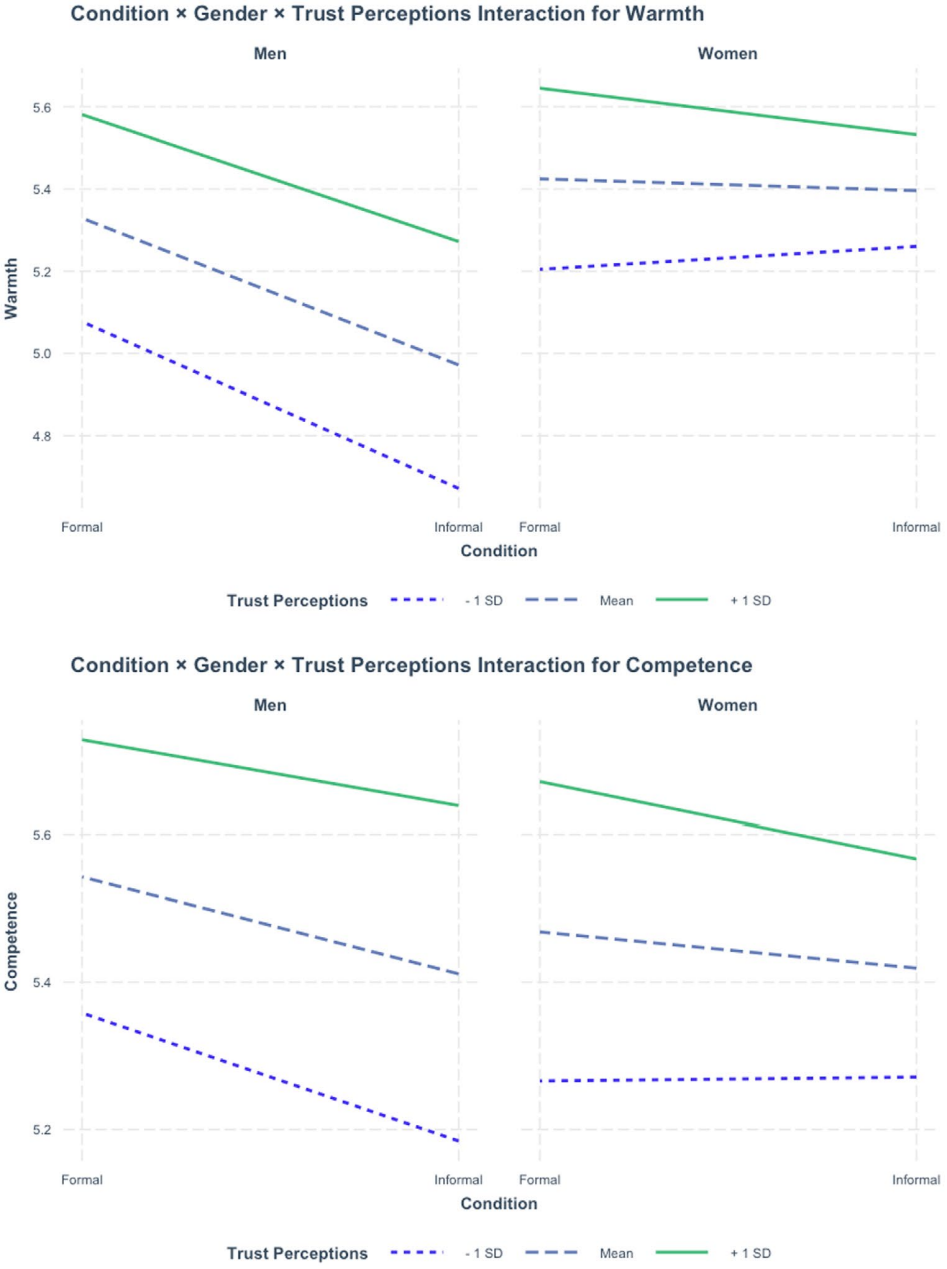
Three‐way interaction effect results.

Exploratory, three‐way interaction effects of Gender × Self‐Presentation × Trust Perceptions were statistically significant for warmth [*F*(1, 3004.8) = 5.46, *p* = .020] and competence [*F*(1, 2917.10) = 3.91, *p* = .048]. Figure 2 displays these patterns, which suggest people typically perceive physicians who are men as warmer and more competent when they wear formal attire compared to informal attire (this occurs regardless of whether people had low, average, or high trust perceptions in physicians, in general). For women physicians, however, the benefits of formal attire are moderated by participants' baseline trust perceptions. Among participants with average and high trust in physicians, formal attire enhances perceptions of women physicians similarly to physicians who are men. Among low‐trust participants, however, formally dressed women physicians are rated as less warm than informally dressed women physicians, while formally dressed men physicians maintain their warmth advantage. For competence, the interaction is somewhat attenuated, with formal attire showing consistent benefits for women physicians at average and high trust levels, but minimal differentiation at low‐trust levels. Altogether, these effects suggest formal attire enhances perceptions of physicians who are men across participant trust levels, while for physicians who are women, the effect of formal attire on perceptions depends on participants' trust levels, with formal attire showing reduced benefits among low‐trust participants.

## DISCUSSION

In the present work, we evaluated the link between physician self‐presentation (e.g. attire) and demographics (e.g. gender) on warmth and competence perceptions, including their joint impact on such attitudinal judgements. We observed that formally dressed physicians were perceived as warmer and more competent than informally dressed physicians; in general, women physicians were rated as warmer than men physicians. Interaction effects suggested formal attire enhanced perceptions of men versus women physicians, and existing trust in physicians moderated how people perceived physicians who are women more than physicians who are men. Therefore, we provide critical evidence to suggest nonverbal self‐presentation characteristics can systematically impact how people think and feel about physicians.

The results of this study are consistent with several conclusions from previous studies. For example, we replicate the idea that physicians in formal attire are perceived as more competent than those in informal attire (Ahmed et al., 2020; Petrilli et al., 2018; Rehman et al., 2005). However, we extend this work in a critical way by further explicating this relationship via physician gender and patient trust in physicians. Patient histories with physicians and medicine as an institution matter (Washington, 2006), and the current work suggests accounting for aspects of such histories via how much people trust physicians can provide important nuance to the relationship between nonverbal self‐presentation, gender and person‐perceptions.

Perhaps our most intriguing finding was the three‐way interaction involving trust in physicians. The evidence suggested patients with lower trust in physicians and the medical profession respond differently to women physicians in formal attire, rating them as slightly less warm compared to those in informal attire, as described in the trend lines for figure 2. This counter‐intuitive finding suggests that for some patients, formal medical attire for women physicians might trigger stereotypes about coldness or distance (Madanay et al., 2025), particularly among those already sceptical of medicine as an institution. This finding has important implications for understanding how patient experiences and attitudes shape their interpretations of physician self‐presentation: The same nonverbal indicators can be interpreted differently depending on both the physician's gender and the patient's pre‐existing relationship with medicine. Our findings emphasize the importance of considering patient/perceiver characteristics—specifically, their trust in institutions—when examining impression formation. The fact that low‐trust perceivers respond differently to formally dressed women physicians suggests that effective self‐presentation must be understood within the context of the audience's prior experiences and attitudes.

### Practical implications

There are several practical implications of our work in addition to those that have enhanced our understanding of self‐presentation theory and impression formation. Our evidence suggests medical schools and residency programs may consider incorporating lessons related to the impact of nonverbal self‐presentation on patient perceptions, particularly focussing on how gender intersects with self‐presentation. Training programs could help physicians develop sensitivity to patient backgrounds and existing trust levels, which may be elusive or difficult to appraise during an initial interaction with a patient. Our findings suggest that understanding a patient's relationship with the medical establishment may inform physicians' self‐presentation choices that could help to facilitate more effective and patient‐centred interpersonal relationships.

### Limitations and future directions

There are limitations in the present study that deserve resolution in the future. First, regarding generalizability, the photographs we chose were not representative of all possible self‐presentations, genders, nor ethnicities. For example, to enhance experimental control, we chose physician photographs for those who presented as White, but future research should investigate how experimentally manipulating race or ethnicity might provide additional nuance to these results. Age is another factor that might impact physician perceptions as well, which should be considered, but we chose photographs for relatively middle‐aged physicians for additional experimental control. Relatedly, our participant population included university students who may not be representative of typical patient populations. Students may have different expectations for physicians' self‐presentation and different levels of experience with the health care system compared to adult patients who may have experienced chronic health conditions or more varied experiences with physicians. Therefore, future studies should consider how participant demographics impact how people think and feel about physicians, and evaluate how actual face‐to‐face assessments of physicians compare to static images. It may also be important to consider perceptions of the same physicians in formal versus informal attire for a more direct comparison of our main research interest.

We only measured two aspects of person perception—warmth and competence, and these are perceptions instead of behavioural measures, which should be evaluated in future work. While the warmth and competence dimensions were theoretically motivated and are some of the most systematically reliable person perception metrics (Fiske, 2018; Fiske et al., 2002), others such as approachability, expertise, cultural competence or patient‐centredness should be considered in future work to see how they are impacted by physician self‐presentation and gender (e.g. for evidence that other dimensions such as morality are orthogonal to warmth and competence, see Goodwin et al., 2014). It might also be instructive to identify how physician perceptions change over time. As patients face positive or negative experiences in the medical system, their perceptions of physicians are likely dynamic as well. Capturing these changes longitudinally in response to key medical events in one's life is an important area of future enquiry. Finally, our reported three‐way interaction regarding self‐presentation, gender and trust is supported by the empirical evidence but still speculative. Future research should measure constructs such as stereotype activation, prior experience with (women) physicians, or attitudes towards professionalism to identify how trust compares to other possible and plausible mechanisms.

## AUTHOR CONTRIBUTIONS

**Hannah Meltser:** Conceptualization; methodology; writing – original draft; writing – review and editing. **David M. Markowitz:** Conceptualization; methodology; formal analysis; project administration; writing – review and editing; writing – original draft; visualization.

## Supporting information


Data S1.

## Data Availability

The data that support the findings of this study are available from the corresponding author upon reasonable request.
