# Supplementary material for: The impact of how physicians self‐present: A study of gender and attire effects on perceived warmth and competence
Source: Br J Health Psychol. 2026 Jan 26;31(1):e70054. doi: 10.1111/bjhp.70054 (PMC12835578; doi:10.1111/bjhp.70054)
Supplement: Supplementary file 1 — Data S1. [file BJHP-31-0-s001.docx]

**Linear Mixed Model Formulae**

warmth_model <- lmer(warmth ~ condition + physician_gender + (1|ResponseId), data = physicians)

warmth_interaction_model <- lmer(warmth ~ condition*physician_gender + (1|ResponseId), data = physicians)

competence_model <- lmer(competence ~ condition + physician_gender + (1|ResponseId), data = physicians)

competence _interaction_model <- lmer(competence ~ condition*physician_gender + (1|ResponseId), data = physicians)
